# The CD40-Autophagy Pathway Is Needed for Host Protection Despite IFN-Γ-Dependent Immunity and CD40 Induces Autophagy via Control of P21 Levels

**DOI:** 10.1371/journal.pone.0014472

**Published:** 2010-12-31

**Authors:** Jose-Andres C. Portillo, Genevieve Okenka, Erin Reed, Angela Subauste, Jennifer Van Grol, Katrin Gentil, Masaaki Komatsu, Keiji Tanaka, Gary Landreth, Beth Levine, Carlos S. Subauste

**Affiliations:** 1 Department of Ophthalmology and Visual Sciences, Case Western Reserve University School of Medicine, Cleveland, Ohio, United States of America; 2 Department of Medicine, Case Western Reserve University School of Medicine, Cleveland, Ohio, United States of America; 3 Department of Neurosciences, Case Western Reserve University School of Medicine, Cleveland, Ohio, United States of America; 4 Department of Internal Medicine, University of Michigan School of Medicine, Ann Arbor, Michigan, United States of America; 5 Department of Pathology, Case Western Reserve University School of Medicine, Cleveland, Ohio, United States of America; 6 Laboratory for Frontier Science, Tokyo Metropolitan Institute of Medical Science, Tokyo, Japan; 7 Department of Internal Medicine, Department of Microbiology, Howard Hughes Medical Institute, University of Texas Southwestern Medical Center, Dallas, Texas, United States of America; INSERM U1016, Institut Cochin, France

## Abstract

Autophagy degrades pathogens *in vitro*. The autophagy gene *Atg5* has been reported to be required for IFN-γ-dependent host protection *in vivo*. However, these protective effects occur independently of autophagosome formation. Thus, the *in vivo* role of classic autophagy in protection conferred by adaptive immunity and how adaptive immunity triggers autophagy are incompletely understood. Employing biochemical, genetic and morphological studies, we found that CD40 upregulates the autophagy molecule Beclin 1 in microglia and triggers killing of *Toxoplasma gondii* dependent on the autophagy machinery. Infected CD40^−/−^ mice failed to upregulate Beclin 1 in microglia/macrophages *in vivo*. Autophagy-deficient Beclin 1^+/−^ mice, mice with deficiency of the autophagy protein Atg7 targeted to microglia/macrophages as well as CD40^−/−^ mice exhibited impaired killing of *T. gondii* and were susceptible to cerebral and ocular toxoplasmosis. Susceptibility to toxoplasmosis occurred despite upregulation of IFN-γ, TNF-α and NOS2, preservation of IFN-γ-induced microglia/macrophage anti-*T. gondii* activity and the generation of anti-*T. gondii* T cell immunity. CD40 upregulated Beclin 1 and triggered killing of *T. gondii* by decreasing protein levels of p21, a molecule that degrades Beclin 1. These studies identified CD40-p21-Beclin 1 as a pathway by which adaptive immunity stimulates autophagy. In addition, they support that autophagy is a mechanism through which CD40-dependent immunity mediates *in vivo* protection and that the CD40-autophagic machinery is needed for host resistance despite IFN-γ.

## Introduction

The lysosomal system is an effector of microbial degradation. Unfortunately, many pathogens including the intracellular protozoan *Toxoplasma gondii* have developed various strategies to avoid lysosomal degradation. Autophagy is a mechanism that can re-route pathogens to lysosomes. Autophagy is a process where an isolation membrane encircles portions of cytosol and organelles leading to the formation of autophagosomes [1], [2]. Fusion between autophagosomes and endosomes-lysosomes culminates in the formation of autolysosomes and degradation of their contents. Autophagy can mediate *in vitro* anti-microbial activity against various pathogens [3], [4], [5], [6], [7]. In the case of *T. gondii* infection, the CD40 – CD154 pathway triggers killing of the parasite within macrophages that is dependent on the autophagy pathway [6], [8]. CD40 is a member of the TNF receptor superfamily expressed on antigen presenting cells as well as on some non-hematopoietic cells, while CD154 (CD40 ligand) is expressed primarily on activated CD4^+^ T cells. The interaction between *T. gondii*-reactive T cells and infected macrophages results in CD40-dependent killing of the parasite through the autophagy pathway [6].

Innate immunity can activate autophagy to mediate host protection *in vivo*. Autophagy protects *Drosophila* against *Listeria monocytogenes* and Vesicular stomatitis virus [9], [10]. In the case of Herpes simplex virus 1 (HSV-1), the virus prevents autophagy by producing the neurovirulence factor ICP34.5 that binds and blocks the effect of the autophagy protein Beclin 1 [11]. A strain of HSV-1 deficient in ICP34.5 does not cause encephalitis in wild type mice but causes disease in mice deficient in PKR, a signaling molecule linked to autophagy [11]. Similarly, in a model of *Salmonella* infection in *Caenorhabditis elegans* and *Dictyostelium discoideum*, autophagy gene inactivation results in increased bacterial replication and decreased animal lifespan [12].

The autophagy gene *ATG5* mediates autophagosome-independent host protection. Studies in mice deficient in *ATG5* in phagocytes revealed that this autophagy gene was required for IFN-γ-mediated *in vivo* host protection likely because *ATG5* was required for the induction of IFN-γ-dependent anti-microbial activity in macrophages [13]. *ATG5* was required for recruitment of the Immunity- Related GTPase (IRG) Irga6 to the parasitophorous vacuole, the damage to this structure and clearance of the parasite [13]. This process occurred independently of classical autophagosome formation. In addition, mice with inactivation of *ATG5* in dendritic cells revealed that this gene promoted the induction of a Th1 response and protection against HSV-1 [14]. The role of *ATG5* in dendritic cells was to enhance MHC II processing of phagocytosed antigens that contain TLR agonists [14]. Processing of antigens for MHC II did not appear to be dependent on canonical autophagy [14]. These studies indicate that *ATG5* regulates IFN-γ-dependent host protection and it does so through a manner that is does not rely on classical autophagy. Thus, the *in vivo* role of autophagy in mediating protection conferred by adaptive immunity is still not completely understood. In addition, it is unclear how adaptive immunity activates autophagy.

*T. gondii* can cause disease that manifests as cerebral and/or ocular toxoplasmosis. IFN-γ, TNF-α and their downstream effector molecule NOS2 are considered the mediators of resistance against ocular and cerebral toxoplasmosis [15], [16], [17], [18], [19], [20]. We report herein that despite the induction of pathogen-reactive T cells, IFN-γ, TNF-α, NOS2, and the preservation of IFN-γ-induced antimicrobial activity in microglia/macrophages, adaptive immunity still required the autophagic pathway to confer *in vivo* protection against cerebral and ocular toxoplasmosis. CD40 upregulates Beclin 1, triggers autophagy and killing of *T. gondii* in microglia independently of IFN-γ, nitric oxide and Irga6. This pathway is required for host protection since mice deficient in Beclin 1, mice with defective expression of Atg7 in microglia/macrophages and CD40^−/−^ mice exhibit impaired autophagy protein-dependent killing of *T. gondii* and are susceptible to cerebral and ocular toxoplasmosis despite induction of *T. gondii*-specific T cell response, upregulation of IFN-γ, TNF-α and NOS2 and unimpaired IFN-γ-induced anti-microbial activity in microglia/macrophages. We also identified a pathway whereby adaptive immunity enhances autophagy. CD40 down-regulates p21 leading to increased levels of Beclin 1 and enhanced autophagy.

## Results

### CD40^−/−^ mice are susceptible to ocular toxoplasmosis and toxoplasmic encephalitis despite upregulation of IFN-γ, TNF-α and NOS2

**Figure 1:**
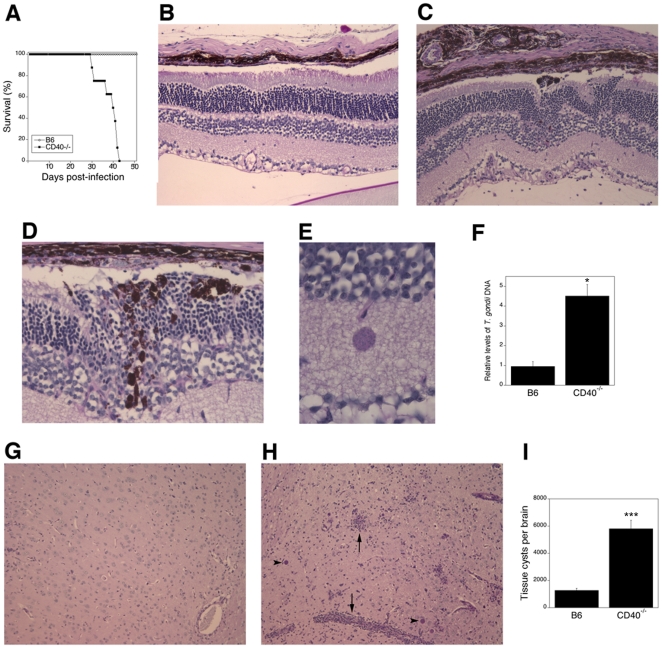
CD40^−/−^ mice are susceptible to the chronic phase of *T. gondii* infection. B6 and CD40^−/−^ mice were infected with tissue cysts of the ME49 strain of *T. gondii*. *A*, Survival was monitored daily. Data shown are from groups containing 8 mice. *B–I*, B6 and CD40^−/−^ mice were euthanized at 4 weeks post-infection. *B*, Eyes from infected B6 mice revealed mild histopathology. *C*–*E*, Eyes from infected CD40^−/−^ mice revealed disruption of retinal architecture, more prominent inflammation (*C*), invasion of the retina by retinal pigmented epithelial cells (*D*) and occasional tissue cysts (*E*). Periodic Acid Schiff-Hematoxylin (PASH); *B*, *C* X200; *D* X400; *E* X1,000. *F*, CD40^−/−^ mice exhibit higher parasite load in the eye than B6 mice. DNA was isolated from eyes. Levels of the *T. gondii B1* gene were examined using quantitative PCR. Each group contained 5 mice. Results are shown as the mean ± SEM. *G*, Brains from infected B6 mice showed slight inflammation. *H*, Brains from infected CD40^−/−^ mice show prominent areas of inflammation (arrow) and numerous tissue cysts (arrow head). PASH X100. *I*, CD40^−/−^ mice exhibit higher numbers of tissue cysts in the brain than B6 mice. Each group contained 5 mice. Results are representative of 4 independent experiments. *≤0.05; ***p≤0.001.

B6 and CD40^−/−^ mice were infected with *T. gondii*. As shown in Figure 1A, CD40^−/−^ died during the chronic phase of infection between weeks 4 to 6 while B6 mice survived at least 11 weeks (p<0.001). We conducted histopathologic studies of the eye and brain. The histological features of the choroid and retina from uninfected B6 and CD40^−/−^ mice were similar. At 4 weeks post-infection, infected B6 mice revealed mild histopathologic changes in the retina and choroid (Figure 1B). In contrast, CD40^−/−^ mice revealed more pronounced histopathology (p<0.01) that included distortion of the retinal architecture, hypertrophy of retinal pigmented epithelial cells, invasion of the retina by these cells, inflammatory infiltrates as well as occasional tissue cysts (Figure 1C–E). Next, we examined the parasite load to determine if worse histopathology in CD40^−/−^ mice was linked to inability to control the parasite. Compared to B6 mice, CD40^−/−^ mice exhibited a significantly higher parasite load in the eye as assessed by quantification of parasite DNA (Figure 1F) (p<0.02).

Histopathologic studies of the brain at 4 weeks post-infection showed that CD40^−/−^ mice had more extensive parenchymal inflammatory foci, perivascular cuffing and more numerous tissue cysts than infected B6 mice (Figure 1G, H). Histopathologic changes were more pronounced in CD40^−/−^ mice than in B6 mice (p<0.01). A significantly higher parasite load in the brain as assessed by the number of tissue cysts accompanied worsened histopathology in CD40^−/−^ mice (Figure 1I) (p<0.001). CD40^−/−^mice also exhibited increased parasite load and more pronounced histopathology at 2 weeks post-infection (not shown). Thus, CD40 is critical for control of ocular and cerebral toxoplasmosis. These results are similar to those obtained with CD154^−/−^ mice [21] and indicate that in contrast to studies with *Mycobacterium tuberculosis* [22], both components of the CD40 – CD154 pathway are critical for resistance against the chronic phase of *T. gondii* infection.

**Figure 2:**
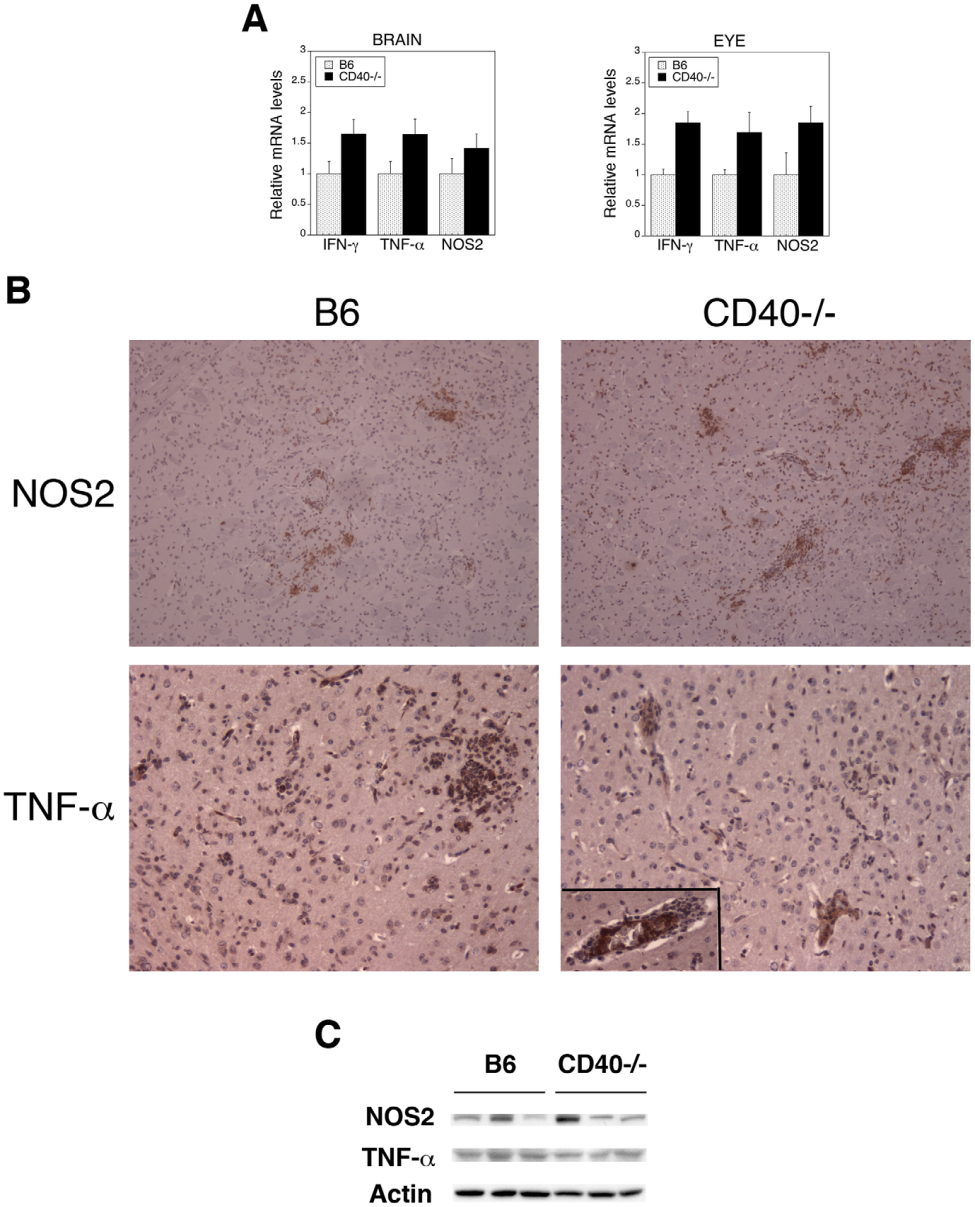
*T. gondii*-infected CD40^−/−^ mice exhibit upregulation of IFN-γ, TNF-α and NOS2 in the eye and brain. B6 and CD40^−/−^ mice were euthanized 4 weeks after infection with tissue cysts of the ME49 strain of *T. gondii*. *A*, Levels of IFN-γ, TNF-α and NOS2 mRNA in eyes and brains were examined using quantitative PCR and normalized against the levels of 18s rRNA. Each group contained 5 mice. Results are shown as the mean ± SEM. *B*, Brains were subjected to immunohistochemistry for NOS2 (X100) and TNF-α (X200). TNF-α was detected not only in the brain parenchyma but also within blood vessel walls in both B6 and CD40^−/−^ mice (inset). *C*, Brains were lysed and subjected to immunoblot for NOS2, TNF-α and actin. Each lane represents one mouse. Results are representative of 3 to 4 independent experiments.

Next, we examined whether susceptibility to toxoplasmosis in CD40^−/−^ mice could be explained by impaired expression of IFN-γ, TNF-α and NOS2. Compared to uninfected mice, *T. gondii* infected animals exhibited at least a 2-log increase in mRNA levels of these genes (not shown) and eyes and brains from both infected CD40^−/−^ and infected B6 mice exhibited upregulation of mRNA of these genes at 2 and 4 weeks post-infection (Figure 2A and not shown). Immunohistochemical studies of the brain and immunoblot of brain lysates at 4 weeks post-infection revealed protein expression of NOS2 and TNF-α in both CD40^−/−^ and control mice (Figure 2B and 2C). Thus, CD40^−/−^ mice are susceptible to ocular and cerebral toxoplasmosis despite upregulation of key mediators of host protection: IFN-γ, TNF-α and NOS2.

### CD40 upregulates autophagy proteins and stimulates autophagy

**Figure 3:**
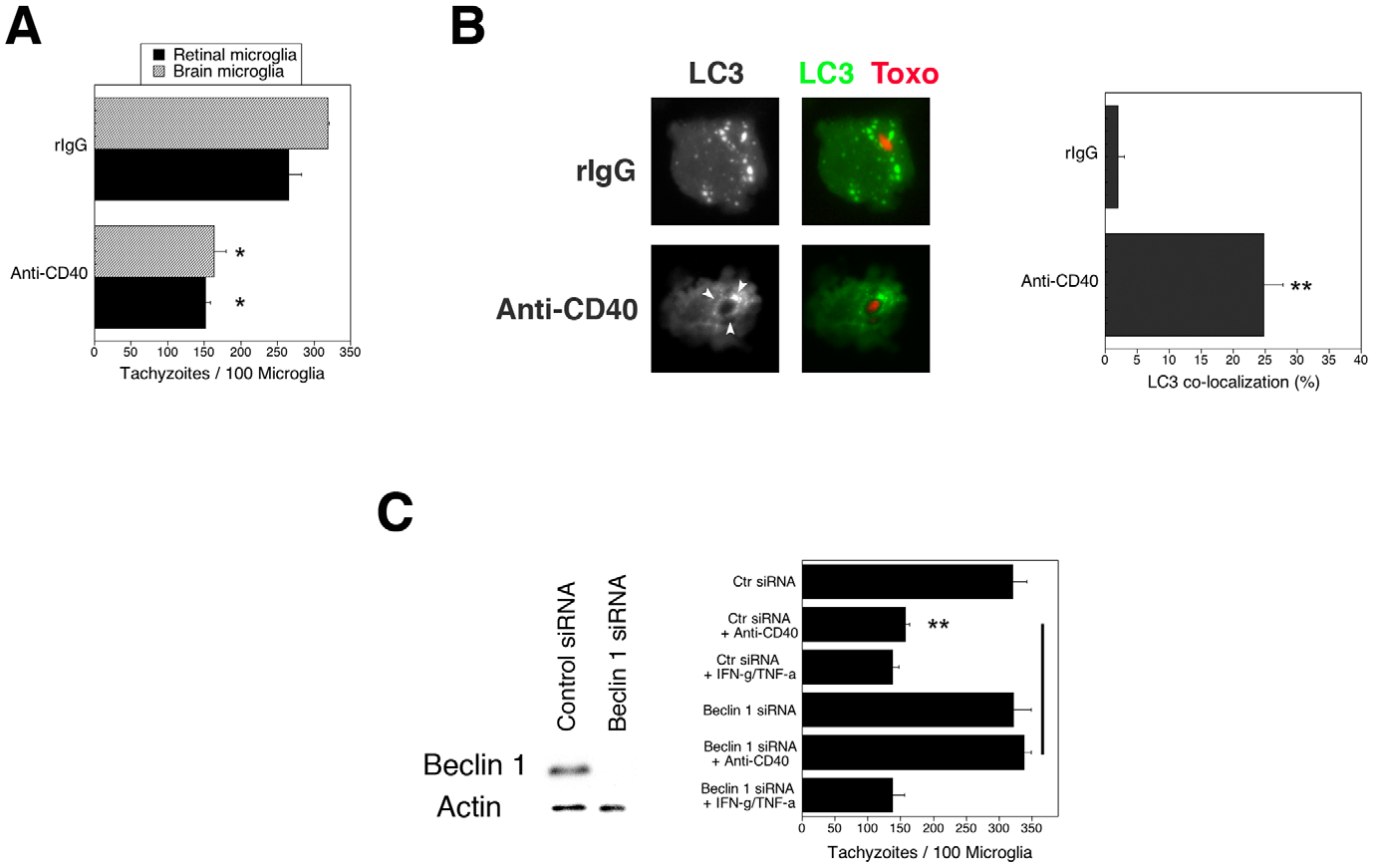
Autophagy mediates anti-*T. gondii* activity induced by CD40 in microglia. *A*, Primary retinal and brain microglia obtained from B6 mice were incubated with a stimulatory anti-CD40 or control mAb, or with IFN-γ/TNF-α followed by infection with tachyzoites of RH *T. gondii*. The numbers of tachyzoites per 100 microglia were determined at 18 h post-infection. *B*, BV-2 cells were transfected with LC3-EGFP followed by incubation with anti-CD40 or control mAb. Cells were infected with RFP-*T. gondii*. Cells were examined at 5 h to determine the percentages of cells where LC3 accumulated around the parasites (arrow head). *C*, BV-2 cells were transfected with control siRNA or siRNA against Beclin 1. Protein expression of Beclin 1 and actin were examined by immunoblot. BV-2 cells transfected with control siRNA or siRNA against Beclin 1 were incubated with a stimulatory anti-CD40 mAb, control mAb or IFN-γ/TNF-α followed by RH *T. gondii* infection. Results are representative of 3 independent experiments. *≤0.05; **p≤0.01.

Microglia are considered central mediators of resistance against *T. gondii* in neural tissue [16]. Microglia were incubated with a stimulatory anti-CD40 or control mAb. Primary brain or retinal microglia treated with a stimulatory anti-CD40 mAb exhibited a significant lower parasite load than control microglia (p<0.02) (Figure 3A). Similar results were obtained with 2 mouse microglia cell lines (BV-2 and N9) or by treating cells with mouse CD154 (not shown). Anti-*T. gondii* activity induced by CD40 was not impaired by a neutralizing anti-IFN-γ mAb, or NMA, an inhibitor of NOS2 (p>0.5) (Figure S1A and S1B). In contrast, anti-IFN-γ mAb or NMA blocked the anti-*T. gondii* activity induced by IFN-γ/TNF-α (p<0.001). The autophagy pathway was required for CD40-induced anti-*T. gondii* activity in microglia because CD40 stimulation caused recruitment of the autophagy marker LC3 around the parasite (Figure 3B) and silencing of Beclin 1 using siRNA ablated CD40-induced anti-*T. gondii* activity (p<0.01) (Figure 3C). In contrast, silencing of Beclin 1 had no effect on IFN-γ/TNF-α induced anti-*T. gondii* activity. The role of the autophagy pathway was also studied in primary microglia and macrophages. Treatment of microglia with the autophagy inhibitor 3-methyl adenine (3-MA) inhibited toxoplasmacidal activity induced by CD40 stimulation (p<0.01) (Figure S2A). Silencing of Atg5 prevented CD40 stimulation from decreasing the parasite load in primary macrophages and microglia (p<0.01) (Figures S2B and S2C). Similarly, silencing of Atg5 in BV-2 cells ablated toxoplasmacidal activity induced by CD40 (p<0.01) (Figure S2D).

Atg5 mediates anti-*T. gondii* activity in IFN-γ-activated macrophages independent of autophagosome formation [13]. IFN-γ induces killing of type II strains of the parasite through Atg5-dependent recruitment of Irga6 to the membrane of the parasitophorous vacuole [13]. We further determined whether the mechanism of anti-microbial activity triggered by CD40 is distinct from that utilized by IFN-γ. Figure S3A shows that CD40 induced anti-microbial activity against not only a type I strain of *T. gondii* (RH) but also against a type II strain (ME49). Knockdown of Beclin 1 ablated the anti-*T. gondii* activity against a type II strain of the parasite induced by CD40 but had no effect on anti-*T. gondii* activity mediated by IFN-γ (Figure S3B). Importantly, knockdown of Irga6 did not impair CD40-induced killing of *T. gondii* while it inhibited the effect of IFN-γ (Figure S3C). Taken together, these studies indicate that CD40 induces entrapment of *T. gondii* by autophagosomes and triggers anti-*T. gondii* activity in retinal and brain microglia through the autophagy pathway, a process that is distinct from that utilized by IFN-γ since CD40 not only acts independently of this cytokine but also independently of Irga6 and nitric oxide. Moreover, CD40 but not IFN-γ requires Beclin 1, an autophagy protein that controls autophagosome formation [23].

**Figure 4:**
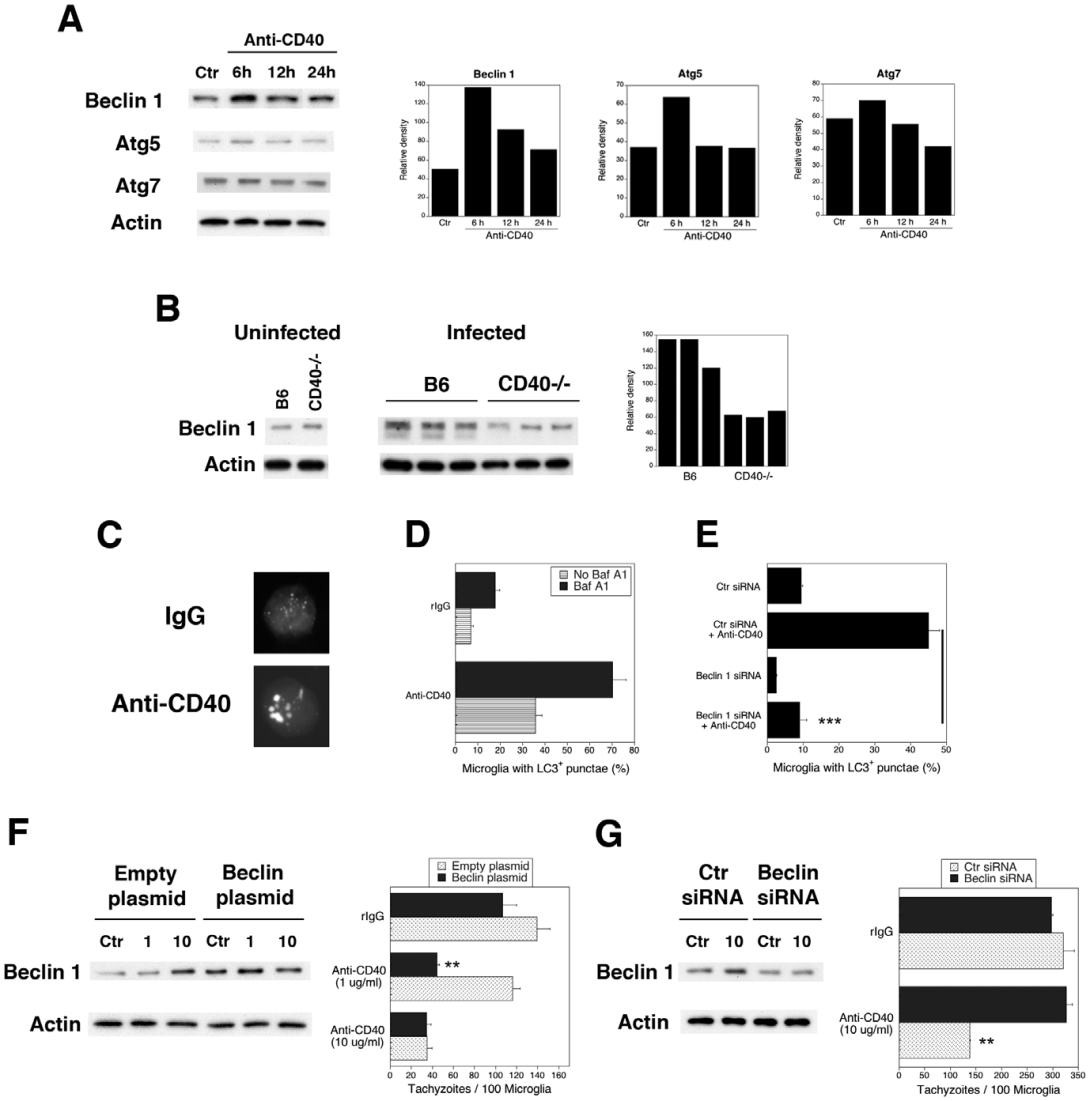
CD40 upregulates autophagy protein expression and leads to increased autophagy. *A*, Primary brain microglia from B6 mice were incubated with anti-CD40 mAb or control mAb. Beclin 1, Atg5 and Atg7 protein expression were normalized against actin. *B*, Beclin 1 and actin protein expression in purified brain microglia from uninfected B6 and CD40^−/−^ mice or purified brain microglia/macrophages from B6 or CD40^−/−^ mice at 4 weeks post-infection. Each lane represents a pool of 2 mice. *C*, *D*, BV-2 cells transfected with LC3-EGFP were incubated with anti-CD40 mAb or control mAb in the presence or absence of bafilomycin A_1_ (BafA1). *E*, BV-2 cells were transfected with control or Beclin 1 siRNA. After 2 days, cells were transfected with LC3-EGFP followed by incubation with anti-CD40 or control mAb. *F*, BV-2 cells were transfected with Beclin 1-encoding or empty plasmids. Expression of Beclin 1 and actin were examined by immunoblot. Cells were incubated with anti-CD40 mAb (1 or 10 µg/ml) or control mAb followed by infection with tachyzoites of RH *T. gondii*. *G*, BV-2 cells transfected with a suboptimal concentration of Beclin 1 siRNA or control were incubated with anti-CD40 mAb (10 µg/ml) or control mAb followed by RH *T. gondii* infection. Results are representative of 3 to 4 independent experiments. **p≤0.01; ***p≤0.001.

The level of Beclin 1 expression has been linked to autophagic activity [24], [25]. We examined whether CD40 modulates Beclin 1 expression. As shown in Figure 4A, CD40 stimulation caused a rapid increase in Beclin 1 protein expression in primary microglia (2.8-fold increase on average; n = 4). CD40 stimulation also caused an apparently less pronounced upregulation of the Atg5 expression (average 1.7-fold) but did not appear to affect expression of Atg7 (Figure 4A). We examined whether CD40 affects the *in vivo* levels of Beclin 1 during *T. gondii* infection. While we did not detect differences in Beclin 1 protein expression between brain microglia from uninfected B6 and CD40^−/−^ mice, Beclin 1 expression in microglia/macrophages from *T. gondii*-infected B6 mice was higher than in microglia/macrophages from infected CD40^−/−^ mice (Figure 4B).

We determined if CD40 not only increased expression of autophagy protein but also enhanced basal levels of autophagy. BV-2 cells were transfected with LC3-EGFP and were incubated with or without anti-CD40 mAb. CD40 stimulation increased the percentage of microglia with LC3^+^ punctae (Figure 4C, D). An increase in these structures could be due to increased autophagosome formation or decreased degradation. Microglia were treated with bafilomycin A_1_, an inhibitor of vacuolar ATPase, which prevents autophagosome degradation [26]. CD40 stimulation still upregulated LC3^+^ punctae when autophagosome degradation was prevented. CD40 stimulated autophagy through Beclin 1 because knock-down of this autophagy protein inhibited upregulation of LC3^+^ punctae (Figure 4E) (p<0.001). Together, CD40 increases expression of Beclin 1 and Atg5 in microglia and enhances autophagy.

Next, we tested whether changes in the level of autophagy protein expression affect toxoplasmacidal activity induced by CD40. We centered on Beclin 1 given that levels of expression of this protein can affect autophagy. BV-2 cells were transfected with a Beclin 1-encoding plasmid or with empty plasmid (Figure 4F). A suboptimal concentration of anti-CD40 mAb (1 µg/ml) did not affect Beclin 1 expression and did not induce anti-*T. gondii* activity in BV-2 cells transfected with the empty plasmid. In contrast, Beclin 1-transfected BV-2 cells exhibited on average a 2.4-fold increase in Beclin 1 protein expression that was accompanied by anti-*T. gondii* activity even if stimulated with 1 µg/ml of anti-CD40 mAb (Figure 4F). Conversely, BV-2 cells transfected with a suboptimal concentration of Beclin 1 siRNA exhibited on average a 53% reduction in Beclin 1 protein levels and ablated CD40-induced anti-*T. gondii* activity despite treatment with a fully activating dose of anti-CD40 mAb (10 µg/ml) (Figure 4G). Thus, relatively modest changes in Beclin 1 expression modulate CD40-induced anti-*T. gondii* activity.

### CD40 stimulation diminishes p21 expression leading to Beclin 1 upregulation

**Figure 5:**
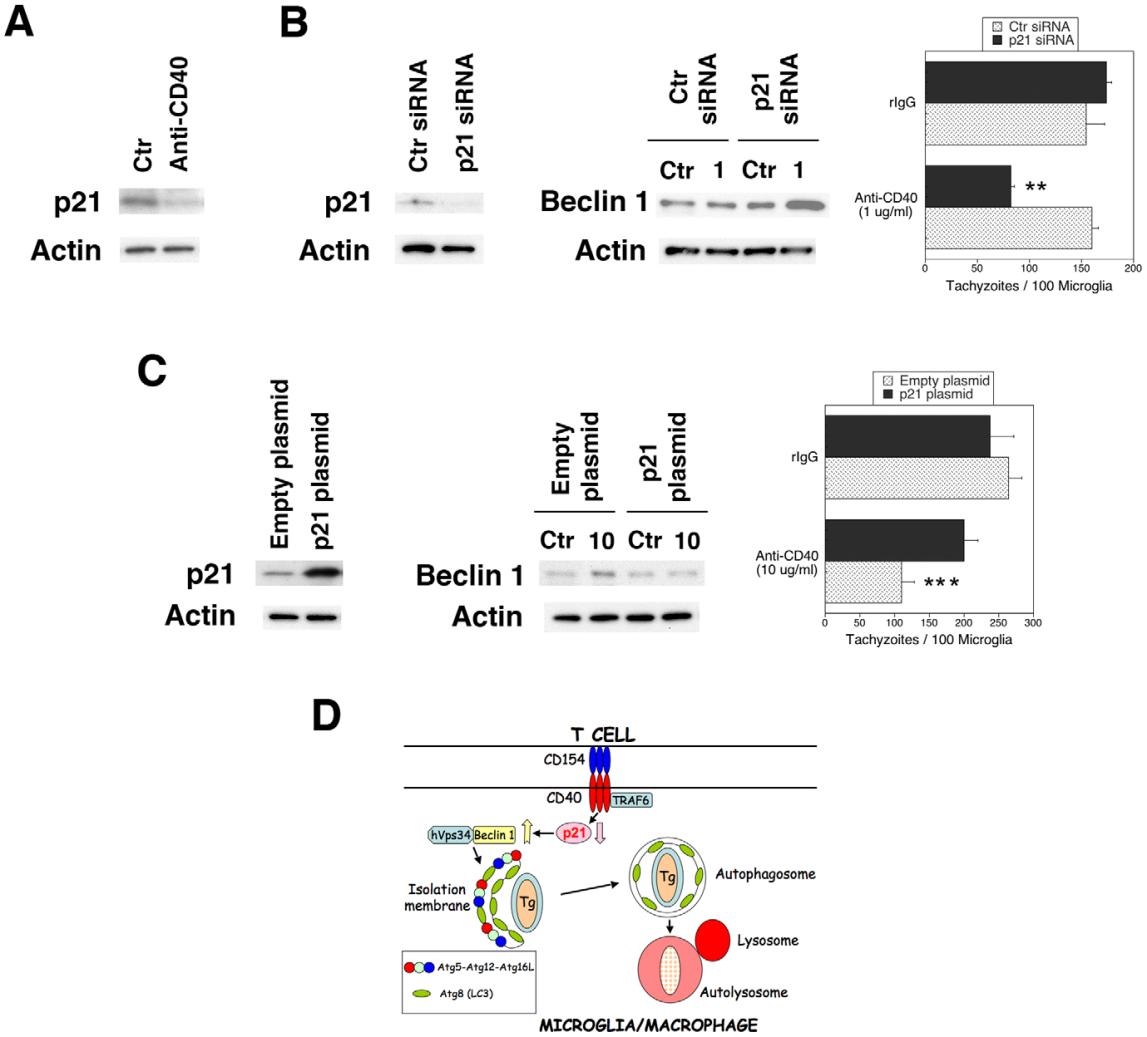
CD40 downregulates p21, increases Beclin 1 expression and autophagic killing of *T. gondii*. *A*, Primary brain microglia from B6 mice were incubated with anti-CD40 mAb or control mAb for 1 h followed by assessment of p21 and actin expression. *B*, BV-2 cells were transfected with control or p21 siRNA. Cells were incubated with a suboptimal concentration of anti-CD40 (1 µg/ml) or control mAb and followed by assessment of Beclin 1 expression. In addition, cells were challenged with RH *T. gondii* and the numbers of tachyzoites per 100 microglia were determined. *C*, BV-2 cells were transfected with p21-encoding or empty plasmids. Cells were incubated with an optimal concentration of anti-CD40 (10 µg/ml) or control mAb and followed by assessment of Beclin 1 expression. Cells were challenged with RH *T. gondii* and the numbers of tachyzoites per 100 microglia were determined. Results are shown as the mean ± SEM and are representative of 3 independent experiments. *D*, Proposed model of the role of p21 in modulation of Beclin 1 expression and activation of *T. gondii* killing induced by CD40. We previously reported that CD40 induces autophagy and killing of *T. gondii* through the adaptor protein TRAF6 [6], [8]. This process would be dependent on down-regulation of p21 that would result in increased levels of Beclin 1 and enhanced autophagy. **p≤0.01; ***p≤0.001.

p21 has been reported to decrease Beclin 1 expression through protein degradation [27]. CD40 upregulates Beclin 1 without affecting Beclin 1 mRNA levels as assessed by real time PCR (Figure S4). Thus, we examined the role of p21 in Beclin 1 upregulation caused by CD40. CD40 stimulation of primary microglia and BV-2 cells caused a rapid decrease in p21 levels (Figure 5A). In addition, knockdown of p21 by transfection with siRNA induced Beclin 1 upregulation in BV-2 cells incubated with a sub-optimal concentration of anti-CD40 mAb (Figure 5B). In contrast, p21 overexpression by transfection with a p21-encoding plasmid prevented CD40 from increasing Beclin 1 levels (Figure 5C). The effects of p21 were of functional relevance because knockdown of p21 promoted anti-*T. gondii* activity in microglia stimulated with a suboptimal concentration of anti-CD40 mAb (Figure 5B) while overexpression of p21 inhibited anti-*T. gondii* activity induced by CD40 (Figure 5C) (p<0.004). These results indicate that p21 regulates Beclin 1 expression and autophagy protein-dependent killing of *T. gondii* in CD40-stimulated microglia (Figure 5D).

### Autophagy-deficient mice are susceptible to ocular toxoplasmosis and toxoplasmic encephalitis

**Figure 6:**
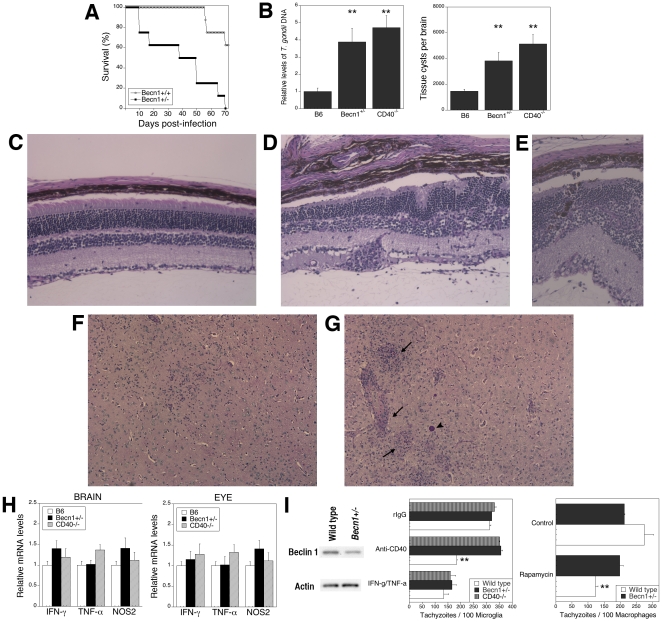
Autophagy-deficient mice are susceptible to cerebral and ocular toxoplasmosis. *Becn1* ^+/−^ and littermate control mice (B6) were infected with ME49 *T. gondii*. *A*, Survival was monitored daily. Data shown are from groups containing 8 mice. *B*, Mice (5 mice per group) were euthanized at 4 weeks post-infection. DNA was isolated from eyes and the levels of the *T. gondii B1* gene were examined using real time quantitative PCR. Tissue cysts were counted in brain homogenates. *C*, Eyes from infected control mice revealed mild histopathology. *D, E*, Eyes from infected *Becn1* ^+/−^ mice revealed disruption of retinal architecture, more prominent inflammation and invasion of the retina by retinal pigmented epithelial cells (*E*). PASH; X200. *F*, Brains from infected control mice showed slight inflammation. *G*, Brains from infected *Becn1* ^+/−^ mice show prominent areas of inflammation (arrow) and frequent tissue cysts (arrow head). PASH X100. *H,* Levels of IFN-γ, TNF-α and NOS2 mRNA in eyes and brains were examined using real time quantitative PCR and normalized against the levels of 18s rRNA. Each group contained 5 mice. *I*, Beclin 1 and actin protein expression in primary microglia from control and *Becn1* ^+/−^ mice. Primary brain microglia from control and *Becn1* ^+/−^ mice and were incubated with anti-CD40 or control mAb, or with IFN-γ/TNF-α followed by infection with tachyzoites of RH *T. gondii*. Primary bone marrow derived macrophages were obtained from control and *Becn1* ^+/−^ mice and were incubated with or without rapamycin (1 µM) 2 h after infection with tachyzoites of RH *T. gondii*. Results are shown as the mean ± SEM and are representative of 3 independent experiments. **p≤0.01.

Our *in vitro* studies indicate that the levels of Beclin 1 modulate killing of *T. gondii*. Based on these findings, we hypothesized that mice with a partial defect in Beclin 1 would exhibit impaired killing of *T. gondii* and susceptibility to ocular and cerebral toxoplasmosis. We used mice with heterozygous deletion of Beclin 1 (*Becn1* ^+/−^ mice) to begin to test the *in vivo* relevance of the autophagic pathway in protection against cerebral and ocular toxoplasmosis. *Becn1* ^+/−^ mice exhibit diminished Beclin 1 protein levels and reduced autophagy *in vivo* [24]. *Becn1* ^+/−^ mice infected with *T. gondii* exhibited decreased survival compared to littermate controls (*Becn1^+/+^*) (Figure 6A) (p<0.001). Moreover, infected *Becn1* ^+/−^ mice exhibited higher parasite loads in the brain and eye (Figure 6B) (p<0.01). Similar to CD40^−/−^ animals, *Becn1* ^+/−^ mice exhibited more pronounced retinal histopathology (p<0.01) that included distortion of the retinal architecture, hypertrophy of retinal pigmented epithelial cells, retinal invasion by these cells and inflammatory infiltrates (Figure 6C–E). In the brain, *Becn1* ^+/−^ mice had more extensive parenchymal inflammatory foci, perivascular cuffing as well as more numerous tissue cysts than control mice (Figure 6F–G) (p<0.01). This increased susceptibility to toxoplasmosis was not explained by diminished expression of IFN-γ, NOS2 and TNF-α in the brain and eye (Figure 6H). Mice with Atg5^−/−^ hematopoietic cells exhibit defects in T cell homeostasis [28]. However, *Becn1* ^+/−^ mice have a partial defect in Beclin 1 expression. Indeed, the phenotypic composition of lymphoid tissue in these mice as well as in CD40^−/−^ mice was similar to that of control mice (Figure S5A). Moreover, these groups of mice exhibited similar percentages of *T. gondii*-reactive IFN-γ- or IL-4-producing CD4^+^ and CD8^+^ T cells as well as equivalent *in vitro* secretion of IFN-γ after stimulation with *T. gondii* lysate antigen (TLA) (Figure S5B and S5C). Next, we studied microglial anti-microbial activity. Microglia from *Becn1* ^+/−^ mice had diminished Beclin 1 protein levels compared to control mice (Figure 6I). In marked contrast to the cytokine studies, microglia from *Becn1* ^+/−^ mice had a defect in killing of *T. gondii* induced by CD40 but not in anti-*T. gondii* activity triggered by IFN-γ/TNF-α (Figure 6I). This defect occurred despite normal expression of CD40 on microglia from *Becn1* ^+/−^ mice (cMFI: Control  = 103±10; *Becn1* ^+/−^  = 112±15). To further confirm that *Becn1^+/−^* mice exhibit defective autophagic killing of *T. gondii*, macrophages from controls and *Becn1^+/−^* mice were incubated with rapamycin, an agent that induces anti-*T. gondii* activity via autophagy [6]. Macrophages from *Becn1^+/−^* mice exhibited impaired killing induced by rapamycin (Figure 6I). Thus, in spite of upregulation of IFN-γ, TNF-α and NOS2, and the development of T cell-mediated immunity against *T. gondii*, mice with defective autophagy and with impaired autophagy protein-dependent killing of the parasite are susceptible to toxoplasmosis.

**Figure 7:**
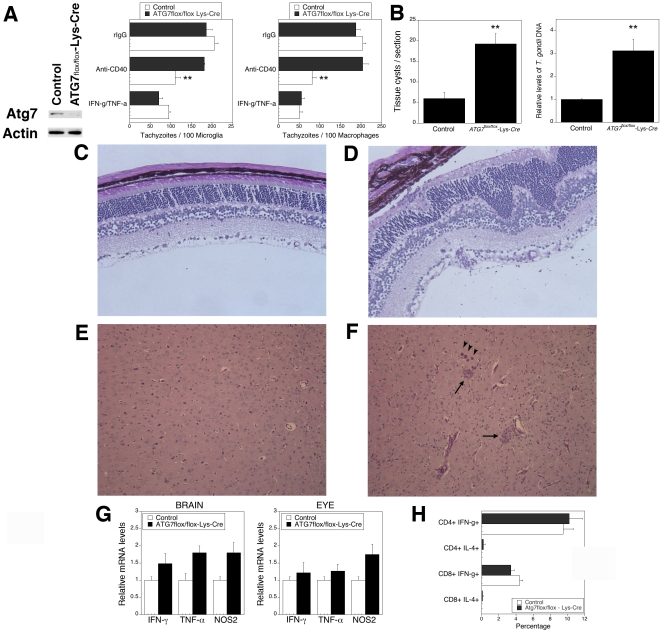
Mice deficient in autophagy in macrophages/microglia are susceptible to toxoplasmosis. *A*, Primary microglia and macrophages were obtained from control and *ATG7^flox/flox^*-Lyz-*Cre* mice. Atg7 and actin expression were assessed by immunoblot. Results shown are those obtained with microglia. Similar findings were observed in primary macrophages. Microglia and macrophages were incubated with a stimulatory anti-CD40 or control mAb, or with IFN-γ/TNF-α followed by infection with tachyzoites of RH *T. gondii*. *B–F*, *ATG7^flox/flox^*-Lyz-*Cre* and control mice were infected with ME49 *T. gondii*. Mice (4–5 mice per group) were euthanized at 3 weeks post-infection. *B*, *ATG7^flox/flox^*-Lyz-*Cre* mice exhibit higher parasite load in the eye and brain than control mice. Numbers of tissue cysts were determined in 100 high power fields. *C, D*, Compared to control mice (*C*), infected *ATG7^flox/flox^*-Lyz-*Cre* mice (*D*) revealed more prominent disruption of retinal architecture and inflammation. PASH; X200. *E*, *F*, Compared to control mice (*E*), infected *ATG7^flox/flox^*-Lyz-*Cre* mice (*F*) show more prominent areas of inflammation (arrow) and frequent tissue cysts (arrow head) in the brain. PASH X100. *G,* Eyes and brains were obtained at 3 weeks post-infection and used to isolate RNA. Levels of IFN-γ, TNF-α and NOS2 mRNA were examined using real time quantitative PCR and normalized against the levels of 18s rRNA. Each group contained 5 mice. *H*, Splenocytes were obtained from control and *ATG7^flox/flox^*-Lyz-*Cre* mice 3 weeks post-infection with ME49 *T. gondii* and were incubated with TLA as described in Materials and Methods. The percentages of CD3^+^ CD4^+^ or CD3^+^ CD8^+^ T cells that became IFN-γ^+^ or IL-4^+^ cells were determined by flow cytometry. Results are shown as the mean ± SEM and are representative of 3 independent experiments. **p≤0.01.

To further evaluate the role of autophagy for *in vivo* control of *T. gondii* in the brain and eye, we conducted experiments using mice where another autophagy gene, *ATG7*, was inactivated in macrophages/microglia. *ATG7* was inactivated in myelomonocytic cells by breeding of *ATG7^flox/flox^* mice [29] with mice that express the Cre recombinase from endogenous lysozyme M locus (Lyz-*Cre*). Indeed, microglia and macrophages from *ATG7^flox/flox^*-Lyz-*Cre* mice had impaired expression of Atg7 (Figure 7A and not shown). Moreover, microglia as well as macrophages from these mice exhibited defective killing of *T. gondii* induced by CD40 but unimpaired parasite killing in response to IFN-γ/TNF-α (Figure 7A). Compared to control mice (*ATG7* ^flox/flox^), *ATG7^flox/flox^*-Lyz-*Cre* mice infected with *T. gondii* were unable to control the parasite in the brain and eye (Figure 7B) (p<0.01) and exhibited more prominent histopathology in these organs (Figure 7C–F) (p<0.01). Similar to the studies with *Becn1^+/−^* mice, *ATG7^flox/flox^*-Lyz-*Cre* mice were susceptible to toxoplasmosis despite upregulation of IFN-γ, TNF-α and NOS2 mRNA (Figure 7G) and the generation of *T. gondii*-specific T cell response (Figure 7H). Thus, mice with defective expression of Atg7 targeted to microglia/macrophages and impaired killing of *T. gondii* exhibit defective *in vivo* control of *T. gondii* in neural tissue.

## Discussion

The cascades by which adaptive immunity activates autophagic killing of an intracellular pathogen and the *in vivo* role of autophagy in protection conferred by adaptive immunity are incompletely understood. We report that CD40 rapidly diminishes the levels of p21, a protein that degrades Beclin 1 [27]. As a result, CD40 causes upregulation of this autophagy protein and stimulates killing of *T. gondii* in microglia dependent on the autophagy pathway. Moreover, mice with diminished expression of Beclin 1 and mice with inactivation of Atg7 in microglia/macrophages are susceptible to cerebral and ocular toxoplasmosis despite upregulation of IFN-γ, TNF-α and NOS2 as well as the induction of T cell-mediated immunity. Susceptibility to the chronic phase of *T. gondii* infection in these mice occurred in the setting of defective autophagy pathway-dependent killing of the parasite while IFN-γ-induced killing of the parasite was unimpaired in these mice. These results contrast the report of autophagosome-independent *in vivo* protection conferred by Atg5, where Atg5 expression in phagocytic cells was required for IFN-γ-mediated killing of the parasite [13]. The findings reported herein strongly support the notion that adaptive immunity utilizes autophagy to confer resistance against an intracellular pathogen because: i) CD40 induced entrapment of the parasite by an LC3^+^ structure; ii) Beclin 1, Atg5 and Atg7 were required for killing of *T. gondii* within CD40-activated microglia and macrophages; iii) Deficiency of two autophagy proteins, Beclin 1 and Atg7, led to susceptibility to toxoplasmosis while leaving unaffected IFN-γ-induced anti-parasite activity and the induction of an IFN-γ-mediated immune response.

One study reported that autophagosomes surround *T. gondii* in primed macrophages [7], [13]. This event followed the disruption of the parasitophorous vacuole membrane raising the possibility that formation of autophagosomes was a response to the presence of disrupted structures within host cells [7], [13]. However, recent work in macrophages did not find evidence of autophagosome formation around the parasite in IFN-γ-activated cells [13]. In addition, studies in astrocytes including work using live-cell imaging revealed that IFN-γ induced killing of *T. gondii* without localization of LC3 to the parasitophorous vacuole [30], [31]. Interestingly, while Atg5 is required in killing of the parasite in IFN-γ-activated cells, this effect occurs in association with recruitment of IRG to the parasitophorous vacuole membrane rather than autophagic entrapment of the parasite [13], [32]. IRG disrupt the parasitophorous vacuole membrane indicating that the anti-*T. gondii* effect of Atg5 in IFN-γ-activated cells is likely dependent on IRG loading onto the parasitophorous vacuole rather that classical autophagy [13]. Our work contrasts to studies in IFN-γ-activated cells. In marked distinction to IFN-γ, CD40 induces toxoplasmacidal activity independently of Irga6 as well as Irgm1 and Irgm3 [33]. Importantly, deficiency of Beclin 1 or Atg7 blocked CD40-induced killing of *T. gondii* while leaving unaffected killing induced by IFN-γ. The different mechanisms used by CD40 and IFN-γ to kill *T. gondii* likely explain the cooperation observed between CD40 and IFN-γ to promote control of the parasite both *in vitro* [34], [35] as well as *in vivo* (this study). Indeed, while IFN-γ, TNF-α and NOS2 are key for resistance against chronic toxoplasmosis, we report that the expression of these mediators does not appear sufficient to prevent ocular and cerebral toxoplasmosis since CD40 as well as the autophagy proteins Beclin 1 and Atg7 are required for protection against the disease.

The autophagic machinery can enhance presentation of certain antigens [14], [36] and can promote the generation of a Th1 response [14], [37]. Our studies indicate that a T cell response against *T. gondii* can develop in mice with defective autophagy supporting the existence of another mechanism by which autophagy promotes resistance against *T. gondii*. Indeed, the studies with *ATG7^flox/flox^*-Lyz-*Cre* mice provide an *in vivo* correlate to our *in vitro* studies on autophagic killing of *T. gondii* in microglia/macrophages and indicate that autophagy in those cells is likely required for resistance against toxoplasmosis in neural tissue.

Beclin 1 plays a central role in autophagy by promoting the formation of autophagosomes [23]. Our studies identified CD40 as a regulator of Beclin 1 since engagement of CD40 resulted in a rapid increase in Beclin 1 expression and enhanced autophagy. Changes in Beclin 1 levels were functionally relevant because a moderate increase in Beclin 1 expression markedly facilitated CD40-induced killing of *T. gondii* while a moderate decrease in Beclin 1 expression profoundly inhibited this response. Moreover, *T. gondii*-infected CD40 deficient mice had lower Beclin 1 levels in their brain microglia/macrophages and Beclin 1-deficient mice were susceptible to cerebral and ocular toxoplasmosis. Studies in cancer and neurodegeneration also support the relevance of Beclin 1 levels. Various cancer cells exhibit decreased Beclin 1 levels and the anti-cancer agent tamoxifen increases expression of this protein [25], [38]. Brain injury appears to upregulate Beclin 1 [39]. In contrast, Beclin 1 is deficient in Alzheimer's dementia and this deficiency impairs neuronal autophagy promoting neurodegeneration [40]. In this model, lentiviral-induced overexpression of Beclin 1 reduces amyloid β pathology [40]. Thus, upregulation of Beclin 1 may drive not only host protection in the CNS but may also activate autophagy as a homeostatic mechanism to reduce cellular damage.

Beclin 1 is not only targeted by CD40-dependent adaptive immunity to stimulate autophagy but can also be targeted by a pathogen to impair autophagy. For example, neurovirulent HSV-1 and HIV-1 encode an ICP34.5 protein and Nef respectively that bind Beclin 1 and block autophagy [11], [41]. The influenza M2 protein blocks autophagosome maturation, an effect that may be mediated through interference with Beclin 1 [42]. Together, these studies and our work indicate that Beclin 1 is an important regulator of autophagy during host-pathogen interaction.

Our results indicate that p21 acts as a link between CD40 and Beclin 1 upregulation and stimulation of autophagy. p21 is not only an inhibitor of cyclin-dependent kinases but it also diminishes expression of Beclin 1 and Atg5 [27]. C_2_-ceramide inhibits autophagy in mouse embryonal fibroblasts because it triggers p21-dependent protein degradation of Beclin 1 and Atg5 [27]. Prior studies addressed the effects of CD40 on p21 expression in B cells. CD40 down-regulates p21 in WEHI 231 immature B lymphoma cells treated with anti-IgM antibody [43]. The discordant results on the effects of CD40 on p21 expression reported in other studies may be explained by differences in the stages of B cell development [44]. We report that CD40 stimulation rapidly reduced p21 protein expression in microglia. Moreover, knockdown of p21 in microglia promoted upregulation of Beclin 1 and autophagic killing of *T. gondii* in response to CD40 stimulation while overexpression of p21 impaired the ability of CD40 to upregulate Beclin 1 and kill *T. gondii*.

Mouse models of *T. gondii* infection are used to study immunity against the parasite. However, these models do not fully mimic the immune response in humans. While IFN-γ is indispensable to control the parasite in mice, mechanisms of resistance against *T. gondii* that do not rely on IFN-γ are likely more effective in humans. Children with an autosomal dominant defect in IFN-γR1 that causes a deletion in the STAT1 binding site do not develop disease when infected with *T. gondii* [45]. In contrast, STAT1^−/−^ mice die within 1 week after infection with the parasite [46], [47]. The differences between the immune response in humans and mice also appear to apply to the downstream effectors of IFN-γ, NOS2 and IRG. NOS2 is more tightly regulated in humans than in rodents and the production of nitric oxide appears to be weaker in humans. Although IRGM has been reported to mediate anti-bacterial activity in human macrophages [48], the role of IRG is likely more restricted in humans than in mice. Mice have 23 IRG genes among which *Irgm1*, *Irgm3* and *Irga6* are important mediators of anti-*T. gondii* activity in mouse cells [30], [49], whereas IRG in humans have been reduced to *IRGM* and *IRGC* [50]. CD40 induces killing of *T. gondii* independently of IFN-γ, STAT1, NOS2, Irgm1, Irgm3 and Irga6 [6], [8], [33], [51]. CD40-induced killing of *T. gondii* may be an important contributor to control of *T. gondii* in humans. Defects in the CD40 pathway are relevant to at least 3 groups of patients that develop ocular and/or cerebral toxoplasmosis: patients with X-linked Hyper IgM syndrome who lack functional CD154 [52], newborns since neonatal CD4^+^ T cells exhibit impaired expression of CD154 [53], [54] and CD40 levels are reduced on neonatal dendritic cells [54], and HIV-1^+^ patients because they exhibit a defect in CD154 induction in their CD4^+^ T cells [55], [56].

In conclusion, our studies revealed that immunity can stimulate autophagy by upregulating autophagy proteins in a p21-dependent manner, and the autophagic machinery was required for resistance against cerebral and ocular toxoplasmosis despite the induction of *T. gondii*-reactive T cells, IFN-γ, TNF-α and NOS2. These results shed new light on the spectrum of mechanisms of host protection. Future studies should explore the possibility of modulation of Beclin 1, autophagy and/or CD40 signaling as an approach to improve pathogen eradication.

## Materials and Methods

### Ethics statement

The animal experimentation guidelines of the US Department of Health and Human Services were followed. The study was approved by the Institutional Animal Care and Use Committee of Case Western Reserve University School of Medicine.

### Animals and parasites

C57BL/6, CD40^−/−^ and mice expressing the Cre recombinase from within the lysozyme M locus (Lyz-*Cre* mice) were purchased from Jackson Laboratories and bred at the Animal Resource Center (Case Western Reserve University). *Becn1^+/−^* and *ATG7* ^flox/flox^ mice have been described [24], [29]. *ATG7* ^flox/flox^ mice were bred with Lyz-*Cre* mice. All mice were female, on a B6 background and were 8–12 week old when used for the studies. Each experimental group consisted of 4–8 mice. Mice were infected i.p. with 10 cysts of ME49 strain of *T. gondii* (gift from Dr. George Yap, Brown University). Tachyzoites of the RH, P (gift from Dr. Louis Weiss, Albert Einstein College of Medicine) and PTG strains of *T. gondii* as well as parasites expressing cytoplasmic DsRed (RFP) were maintained as described [6].

### Histopathology and Immunohistochemistry

Unless otherwise stated, mice were anesthetized and perfused with PBS 4 weeks after infection. Four 5 µm sections at different areas of the brains and eyes were stained with periodic acid Schiff hematoxylin (PASH) stain and used to score histopathologic changes using previously described criteria [16], [17]. Tissue cysts were counted in brain homogenates or in tissue sections as previously described [19]. Sections were incubated with anti-NOS2 or anti-TNF-α Ab (Millipore and Abcam respectively) followed by incubation with secondary antibody conjugated to biotin (Jackson ImmunoResearch Laboratories). Sections were resolved using a Vectastain ABC kit (Vector Laboratories).

### Real-time quantitative PCR

RNA was obtained from eyes and brains using the RNeasy kit (QIAGEN). After treatment with DNase (Ambion), 0.5 µg of RNA was reverse transcribed to cDNA with Super-Script III reverse transcriptase (Invitrogen) and oligo(dT)_12–18_ primers. cDNA (2.5 µl) was used as template for RT-PCR using SYBR GREEN PCR Master Mix (Applied Biosystems) and 20 pM (each) primers in 50 ml. Gene expression was assessed using a 7300 Real Time PCR System (Applied Biosystems). Each cDNA sample was run in duplicate. Samples were normalized according to the content of 18S rRNA [55]. Expression of the *T. gondii B1* gene was assessed by real-time quantitative PCR. Genomic DNA was isolated from eyes using a DNeasy kit (QIAGEN) and subjected to real-time PCR using SYBR GREEN PCR Master Mix. Each sample was run in duplicate and normalized against *L32*. Primer sequences for IFN-γ [57], TNF-α [57], NOS2 [58], 18S rRNA [55] and *T. gondii B1* gene [59] were previously described. Primer sequences for Beclin 1 were forward: 5′-GGCCAATAAGATGGGTCTGA-3′, reverse: 5′-GCTGCACACAGTCCA GAAAA– 3′. Primers for the *T. gondii B1* gene have been previously described [59]. Primers for *L32* were: forward 3′-TGTGCAACAAATCTTCACCGTGC-5′;

reverse 3′-GGATTGGTGACTCTGATGGCC-5′.

### Microglia and *in vitro* infection with *T. gondii*

Primary brain and retinal microglia as well as mouse brain microglial cell lines BV-2 and N9 (gifts from Drs. Kalipada Pahan, Univ. of Nebraska and Dr. Jun Tan, Univ. of South Florida respectively) were treated overnight with a stimulatory anti-CD40 mAb (1C10; 1 or 10 µg/ml), isotype control mAb or IFN-γ (100 U/ml; PeproTech) plus TNF-α (250 pg/ml; PeproTech) [51]. Tachyzoites of the RH or ME49 strains of *T. gondii* were used to infect monolayers as described [51]. When indicated, microglia were incubated with *N* ^G^-monomethyl-L-arginine (NMA, 100 µM; Calbiochem) or a neutralizing mAb against mouse IFN-γ (XMG 1.2; 10 µg/ml; eBiosciences) in the presence of anti-CD40 mAb, control mAb or IFN-γ/TNF-α. 3-methyl adenine (3-MA; 10 mM; Sigma-Aldrich) was added to monolayers 1 hour after infection. Parasite replication was assessed by light microscopy [51]. To isolate brain mononuclear cells from infected mice, brains were digested with papain (15 IU/ml)/DNAse (15 µg/ml; Worthington Biochemicals) for 30 min at 37°C. Tissue was dissociated and cells were loaded onto a gradient made with 30% and 60% isotonic Percoll. Gradients were centrifuged at 1000× g for 30 min. Cells present in the interphase were collected and washed. Microglia/macrophages were purified using anti-CD11b-coated magnetic beads (Miltenyi Biotec). This resulted in populations that were >95% F4/80^+^.

### Lentiviral vectors

We constructed an shRNA against mouse *Atg5* using the following oligo pair (sense and antisense strands are underlined while the loop is italicized): forward 5′-TGGATGAGATAACTGAAAGA *TTCAAGAGA* TCTTTCAGTTATCTCATCCTTTTTTC-3′; reverse 5′-TCGAGAAAAAAGGATGA GATAACTGAAAGA *TCTCTTGAA* TCTTTCAGTTATCTCATCCA-3′. An XhoI restriction site was included at the 5′-end of the reverse oligo to facilitate cloning. An shRNA against luciferase was used as control [60]. Oligo pairs were annealed and subcloned into the polylinker region of the pLL3.7 vector [61] (American Type Tissue Collection) followed by sequence verification. To generate lentivirus, we co-transfected pLL3.7 containing shRNA and packaging vectors VSV-G, RSV-REV, pMDL-g/p RRE into 293T cells. Supernatants were collected at 24 and 48 h, passed through a 0.45 µm filter, concentrated by ultracentrifugation and stored at −80°C. Cells were transduced by incubation with lentiviral particles at a MOI of 10∶1 in the presence of polybrene (8 µg/ml). EGFP^+^ cells were sorted by FACS at 4 days post-transduction. Efficiency of gene silencing was determined by immunoblot.

### Transfection of microglia and immunofluorescence

Using an Amaxa nucleofector as previously described [6], BV-2 cells were transfected with either control siRNA, Beclin 1 siRNA [62], p21 siRNA [27], Irga6 siRNA [63] (all from Dharmacon), empty vector, or plasmid that encode Beclin 1 [64], p21 [65] or LC3-EGFP (gift from Tamotsu Yoshimori, National Institute for Basic Biology, Okazaki, Japan). The numbers of LC3^+^ punctae were determined using a Leica DMI 6000 B automated microscope equipped for epifluorescence microscopy. Distinct structures that measure at least 1 µm in diameter were identified as LC3 punctae. Experimental groups had triplicate samples and at least 50 cells per sample were counted. Co-localization of LC3-EGFP around RFP-*T. gondii* was assessed as described [6].

### Immunoblot

Membranes were probed with either antibody to Atg5 (ProteinTech Group Inc), Atg7 (Cell Signaling), Beclin 1 (BD Biosciences), p21 (Santa Cruz Biotechnologies), NOS2 (BD Biosciences), TNF-α (Abcam Inc.), Irga6 (Santa Cruz Biotechnologies) or actin (Santa Cruz Biotechnologies), followed by incubation with secondary Ab conjugated to horseradish peroxidase (Santa Cruz Biotechnologies). Bands were developed using enhanced chemiluminescence. In experiments that assessed the effect of CD40 stimulation on Beclin 1, Atg5, Atg7 and p21 expression, microglia were pre-incubated with IFN-γ for 18 h to increase the percentage of CD40^+^ cells. After extensive washing, microglia were incubated with anti-CD40 or control mAb. Pre-incubation with IFN-γ was never used in studies of CD40-dependent anti-microbial activity.

### Flow cytometry

Cells were stained with anti-CD3, anti-CD4, anti-CD8, anti-CD11b, anti-CD40, anti-CD45R, anti-CD49d (DX5), anti-Ly6G (Gr-1), anti-F4/80 or isotype control mAbs (all from eBiosciences). For detection of intracellular cytokines, splenocytes were incubated with *T. gondii* lysate antigen (TLA; 10 µg/ml) for 48 and 72 h. PMA (1 ng/ml; Sigma Chemical), ionomycin (1 µg/ml; Sigma Chemical) and brefeldin A (10 µg/ml; eBiosciences) were added during the last 5 h of incubation. Cells were permeabilized using IntraPrep permeabilization reagent (Coulter-Immunotech) and stained with anti-IFN-γ or anti-IL-4 mAb (eBiosciences). Cells were analyzed using an LSR II (BD Biosciences).

### Cytokine assays

CD4^+^ T cells were purified from the spleens of infected animals using magnetic beads (Miltenyi). CD4^+^ T cells (1×10^6^/ml) and were incubated with autologous bone marrow derived macrophages (2.5×10^5^/ml) plus either TLA (10 µg/ml) or concanavalin A (5 µg/ml; Sigma Chemical). Supernatants were collected at 48 h. Concentrations of IFN-γ in tissue culture supernatants were determined by enzyme-linked immunosorbent assay (Endogen).

### Statistical analysis

Statistical significance was assessed by Student's *t* test and ANOVA. Mortality curves and histopathologic changes were analyzed using Mann-Whitney *U* test. Differences were considered statistically significant when *P* was <0.05.

## Supporting Information

Figure S1 CD40 induces anti-*T. gondii* activity in microglia independently of IFN-γ and nitric oxide. A, B, Primary brain microglia were incubated with a stimulatory anti-CD40 or control mAb, or with IFN-γ/TNF-α as indicated followed by infection with tachyzoites of RH *T. gondii*. Microglia were also treated with a neutralizing anti-IFN-γ mAb (A) or NMA (B) prior to addition of anti-CD40 mAb or IFN-γ/TNF-α. The numbers of tachyzoites per 100 microglia were determined microscopically at 18 h post-infection. Results are shown as the mean + SEM and are representative of 3 independent experiments. **p<0.01.
(0.11 MB TIF)

Figure S2 Autophagy mediates anti-*T. gondii* activity induced by CD40 in microglia. A, Primary brain microglia from B6 mice were incubated with a stimulatory anti-CD40 or control mAb, or with IFN-γ/TNF-α as indicated. 3-MA or vehicle were added 1 hour after challenge with RH *T. gondii*. The numbers of tachyzoites per 100 microglia were determined microscopically at 18 hours post-infection. B–D, Knockdown of Atg5 abrogates anti-*T. gondii* activity induced by CD40 stimulation. Primary bone marrow-derived macrophages (B), primary brain microglia (C) or BV-2 cells (D) were transduced with EGFP-encoding lentiviral vectors that contained either shRNA against Atg5 or control shRNA. EGFP^+^ cells were sorted 4 days after transduction. Protein expression of Atg5 and actin were examined by immunoblot. Cells were then incubated with a stimulatory anti-CD40 mAb, control mAb or IFN-γ/TNF-α followed by infection with RH *T. gondii*. The numbers of tachyzoites per 100 microglia/macrophages were determined microscopically at 18 h post-infection. Results are shown as the mean + SEM and are representative of 3 independent experiments. **p<0.01.
(0.17 MB TIF)

Figure S3 CD40 induces anti-microbial activity against not only type I but also type II strains of *T. gondii*, and this effect is independent of Irga6. A, BV-2 cells were incubated with a stimulatory anti-CD40 or control mAb followed by challenge with tachyzoites of the RH strain of *T. gondii* (type I strain) or the P strain (ME49 clone) of the parasite (type II strain). The numbers of tachyzoites per 100 microglia were determined microscopically at 18 hours post-infection. B, BV-2 cells were transfected with control siRNA or siRNA against Beclin 1. BV-2 cells transfected with control siRNA or siRNA against Beclin 1 were incubated with a stimulatory anti-CD40 mAb, control mAb or IFN-γ/TNF-α followed by infection with a type II strain of *T. gondii* (P strain). C, BV-2 cells were transfected with control or Irga6 siRNA. Protein expression of Irga6 and actin were examined by immunoblot. Cells were then incubated with a stimulatory anti-CD40 mAb, control mAb or IFN-γ/TNF-α followed by infection with a type II strain of *T. gondii* (P strain). Results are shown as the mean + SEM and are representative of 3 independent experiments. **p<0.01.
(0.15 MB TIF)

Figure S4 CD40 does not affect Beclin 1 mRNA levels. A, Primary brain microglia from B6 mice were incubated with a stimulatory anti-CD40 mAb or control mAb. Serial dilutions of cDNA were used to examine levels of Beclin 1 mRNA by real-time quantitative PCR, which were normalized against the levels of 18s rRNA. B, Four weeks after infection with ME49 *T. gondii*, microglia/macrophages were purified from brains of B6 or CD40^−/−^ mice. Beclin 1 mRNA levels were examined by real-time PCR. Each bar represents a single mouse. Results are shown as the mean + SEM and are representative of 3 independent experiments.
(0.09 MB TIF)

Figure S5 Becn1^+/−^ mice do not have abnormal lymphoid organ phenotypic composition or defective induction of *T. gondii*-specific T cell response. A, Splenocytes were obtained from uninfected control, Becn1^+/−^ and CD40^−/−^ mice. Expression of CD4^+^ T cells (CD3^+^ CD4^+^), CD8^+^ T cells (CD3^+^ CD8^+^), B cells (CD45R^+^), NK cells (CD49d^+^), and granulocytes (Ly6-G^+^) were examined by flow cytometry. B, Splenocytes were obtained from control, Becn1^+/−^ and CD40^−/−^ mice 4 weeks post-infection with ME49 *T. gondii* and were incubated with TLA as described in Materials and Methods. The percentages of CD3^+^ CD^4+^ or CD3^+^ CD8^+^ T cells that became IFN-γ^+^ or IL-4^+^ cells were determined by flow cytometry. C, Purified CD4^+^ T cells were obtained 4 weeks post-infection with ME49 *T. gondii* and were stimulated with macrophages plus TLA or Concanavalin A (Con A). IFN-γ in supernatants was measured by ELISA. Results are shown as the mean + SEM and are representative of 3 independent experiments.
(0.12 MB TIF)
