# Supplementary figures and images for: The CD40-Autophagy Pathway Is Needed for Host Protection Despite IFN-Γ-Dependent Immunity and CD40 Induces Autophagy via Control of P21 Levels
Source: PLoS One. 2010 Dec 31;5(12):e14472. doi: 10.1371/journal.pone.0014472 (PMC3013095; doi:10.1371/journal.pone.0014472)

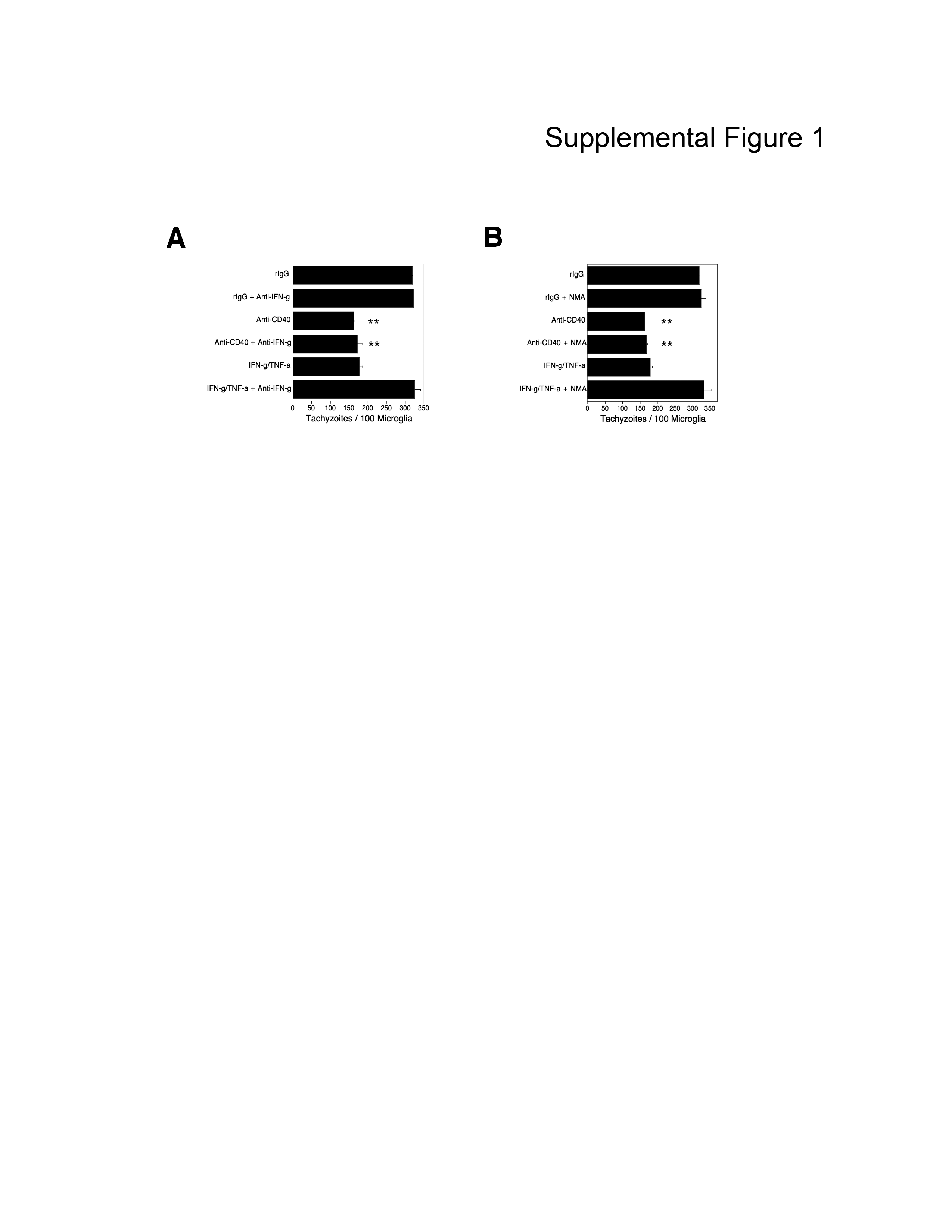

Supplement: Figure S1 — CD40 induces anti-T. gondii activity in microglia independently of IFN-γ and nitric oxide. A, B, Primary brain microglia were incubated with a stimulatory anti-CD40 or control mAb, or with IFN-γ/TNF-α as indicated followed by infection with tachyzoites of RH T. gondii. Microglia were also treated with a neutralizing anti-IFN-γ mAb (A) or NMA (B) prior to addition of anti-CD40 mAb or IFN-γ/TNF-α. The numbers of tachyzoites per 100 microglia were determined microscopically at 18 h post-infection. Results are shown as the mean + SEM and are representative of 3 independent experiments. **p<0.01. (0.11 MB TIF) [file pone.0014472.s001.tif]

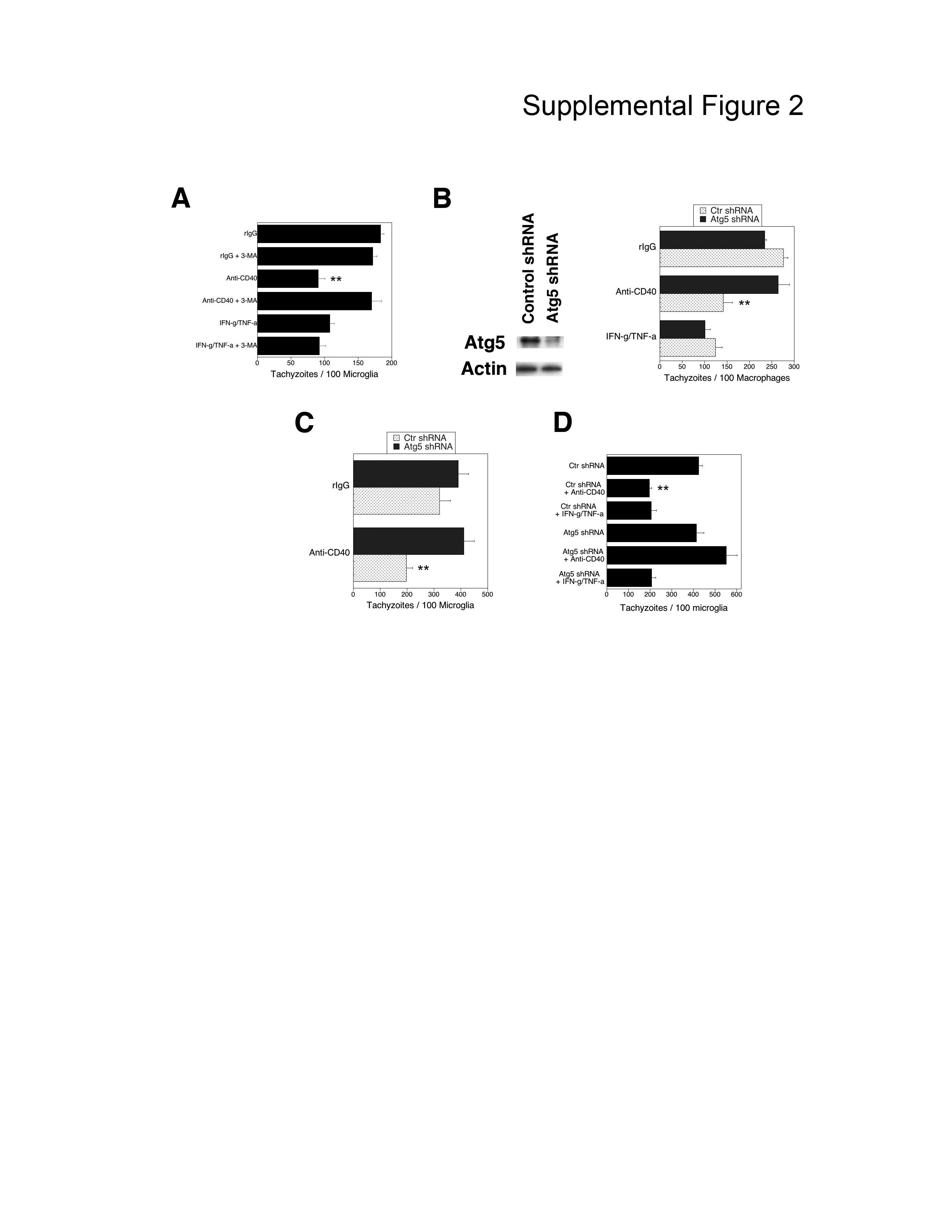

Supplement: Figure S2 — Autophagy mediates anti-T. gondii activity induced by CD40 in microglia. A, Primary brain microglia from B6 mice were incubated with a stimulatory anti-CD40 or control mAb, or with IFN-γ/TNF-α as indicated. 3-MA or vehicle were added 1 hour after challenge with RH T. gondii. The numbers of tachyzoites per 100 microglia were determined microscopically at 18 hours post-infection. B–D, Knockdown of Atg5 abrogates anti-T. gondii activity induced by CD40 stimulation. Primary bone marrow-derived macrophages (B), primary brain microglia (C) or BV-2 cells (D) were transduced with EGFP-encoding lentiviral vectors that contained either shRNA against Atg5 or control shRNA. EGFP+ cells were sorted 4 days after transduction. Protein expression of Atg5 and actin were examined by immunoblot. Cells were then incubated with a stimulatory anti-CD40 mAb, control mAb or IFN-γ/TNF-α followed by infection with RH T. gondii. The numbers of tachyzoites per 100 microglia/macrophages were determined microscopically at 18 h post-infection. Results are shown as the mean + SEM and are representative of 3 independent experiments. **p<0.01. (0.17 MB TIF) [file pone.0014472.s002.tif]

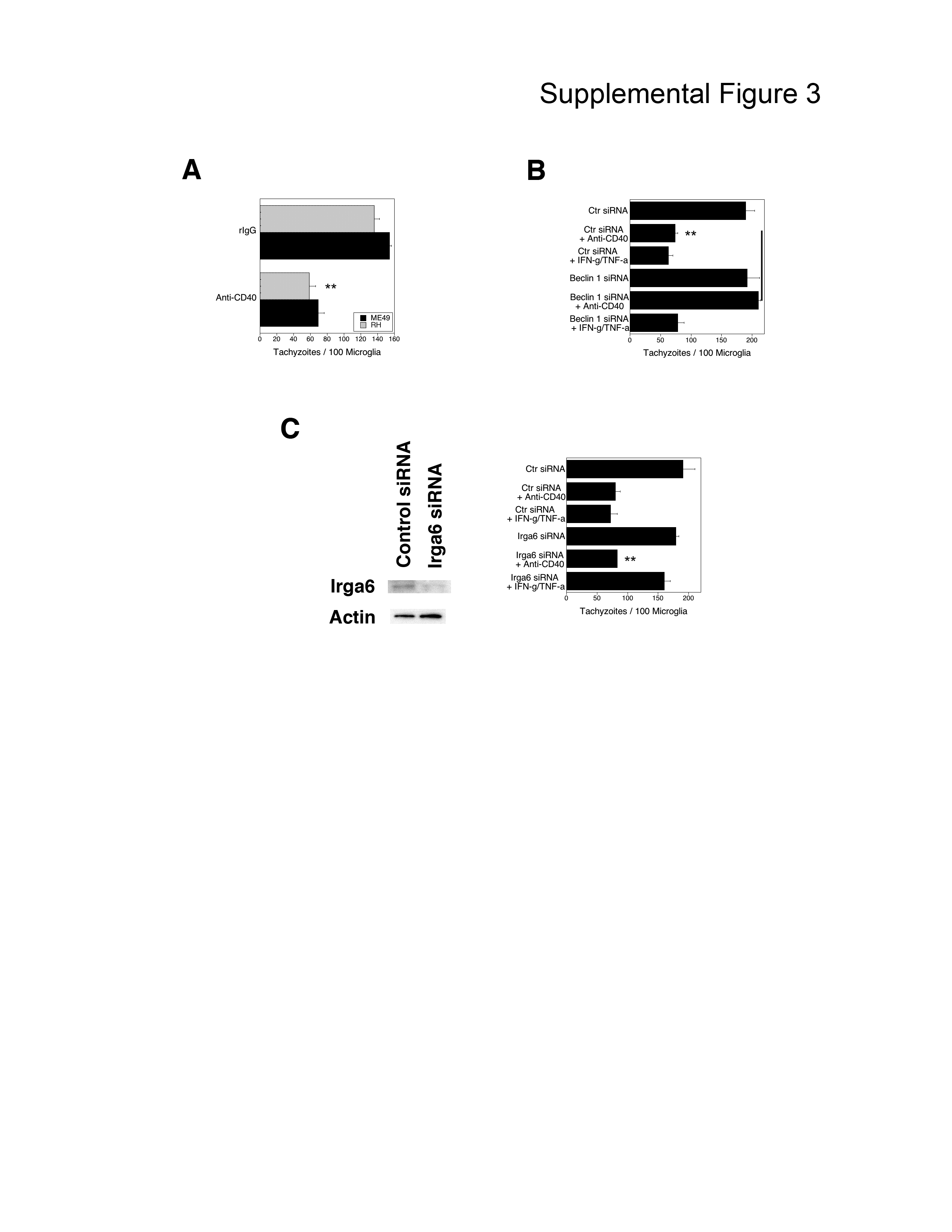

Supplement: Figure S3 — CD40 induces anti-microbial activity against not only type I but also type II strains of T. gondii, and this effect is independent of Irga6. A, BV-2 cells were incubated with a stimulatory anti-CD40 or control mAb followed by challenge with tachyzoites of the RH strain of T. gondii (type I strain) or the P strain (ME49 clone) of the parasite (type II strain). The numbers of tachyzoites per 100 microglia were determined microscopically at 18 hours post-infection. B, BV-2 cells were transfected with control siRNA or siRNA against Beclin 1. BV-2 cells transfected with control siRNA or siRNA against Beclin 1 were incubated with a stimulatory anti-CD40 mAb, control mAb or IFN-γ/TNF-α followed by infection with a type II strain of T. gondii (P strain). C, BV-2 cells were transfected with control or Irga6 siRNA. Protein expression of Irga6 and actin were examined by immunoblot. Cells were then incubated with a stimulatory anti-CD40 mAb, control mAb or IFN-γ/TNF-α followed by infection with a type II strain of T. gondii (P strain). Results are shown as the mean + SEM and are representative of 3 independent experiments. **p<0.01. (0.15 MB TIF) [file pone.0014472.s003.tif]

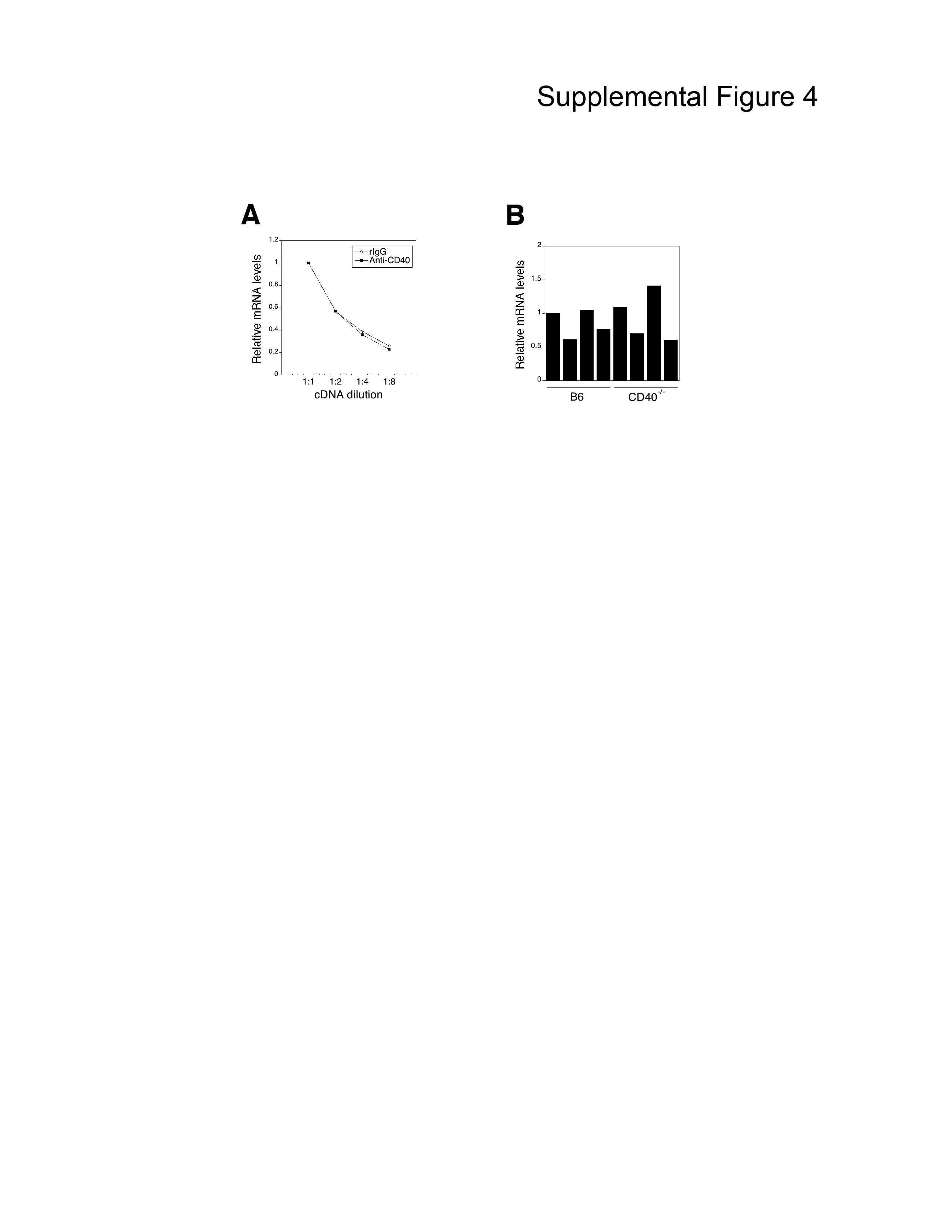

Supplement: Figure S4 — CD40 does not affect Beclin 1 mRNA levels. A, Primary brain microglia from B6 mice were incubated with a stimulatory anti-CD40 mAb or control mAb. Serial dilutions of cDNA were used to examine levels of Beclin 1 mRNA by real-time quantitative PCR, which were normalized against the levels of 18s rRNA. B, Four weeks after infection with ME49 T. gondii, microglia/macrophages were purified from brains of B6 or CD40−/− mice. Beclin 1 mRNA levels were examined by real-time PCR. Each bar represents a single mouse. Results are shown as the mean + SEM and are representative of 3 independent experiments. (0.09 MB TIF) [file pone.0014472.s004.tif]

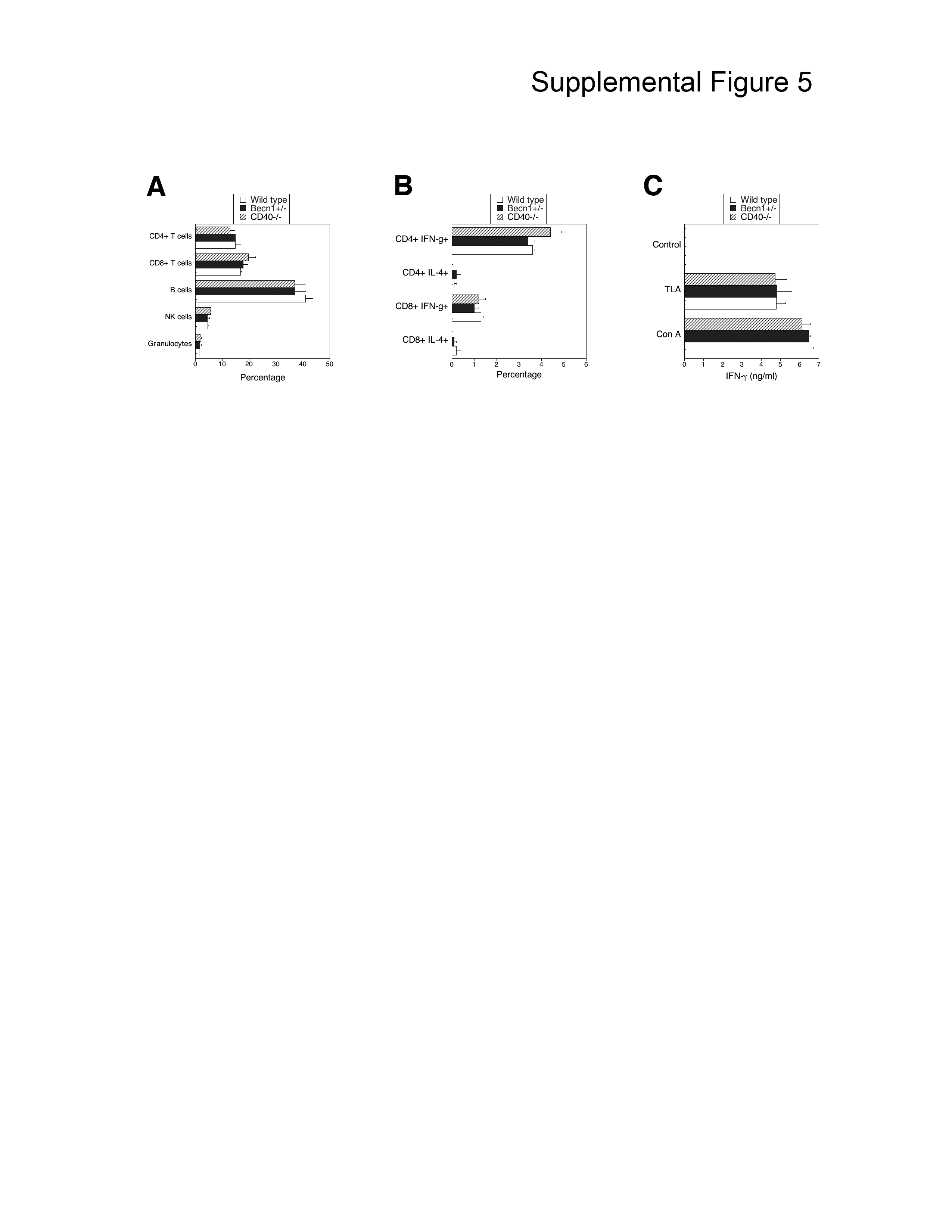

Supplement: Figure S5 — Becn1+/− mice do not have abnormal lymphoid organ phenotypic composition or defective induction of T. gondii-specific T cell response. A, Splenocytes were obtained from uninfected control, Becn1+/− and CD40−/− mice. Expression of CD4+ T cells (CD3+ CD4+), CD8+ T cells (CD3+ CD8+), B cells (CD45R+), NK cells (CD49d+), and granulocytes (Ly6-G+) were examined by flow cytometry. B, Splenocytes were obtained from control, Becn1+/− and CD40−/− mice 4 weeks post-infection with ME49 T. gondii and were incubated with TLA as described in Materials and Methods. The percentages of CD3+ CD4+ or CD3+ CD8+ T cells that became IFN-γ+ or IL-4+ cells were determined by flow cytometry. C, Purified CD4+ T cells were obtained 4 weeks post-infection with ME49 T. gondii and were stimulated with macrophages plus TLA or Concanavalin A (Con A). IFN-γ in supernatants was measured by ELISA. Results are shown as the mean + SEM and are representative of 3 independent experiments. (0.12 MB TIF) [file pone.0014472.s005.tif]
